# MIXCOP–Implementation of a model to assess the time-dependent effects of mixed pesticides on a collembolan population

**DOI:** 10.1016/j.mex.2025.103403

**Published:** 2025-05-28

**Authors:** Alexandra Sybertz, Richard Ottermanns, Martina Roß-Nickoll

**Affiliations:** Institute for Environmental Research, RWTH Aachen University, Germany

**Keywords:** Pesticide mixtures, Impact assessment, Soil organisms, Organism movement, Non-target organisms, MIXCOP

## Abstract

A new simulation model has been created by merging three model approaches, including soil exposure prediction, effect estimation and a population model.

• The aim is to use the overall model to better assess the effects of pesticides on soil organisms.
• The model is called MIXCOP (pesticide MIXtures and their effects on a COllembolan Population) and simulates a population of *Folsomia candida* in a vertical soil column.
• MIXCOP allows to calculate a time- and movement-dependent exposure for the individual animals, as well as the resulting effects. Effects on both adult organisms and juvenile organisms are taken into account. The modular structure including the individual assumptions are explained in more detail.

## Graphical abstract

This graphic was published in advance in Sybertz (2024) [1].

**Figure:**
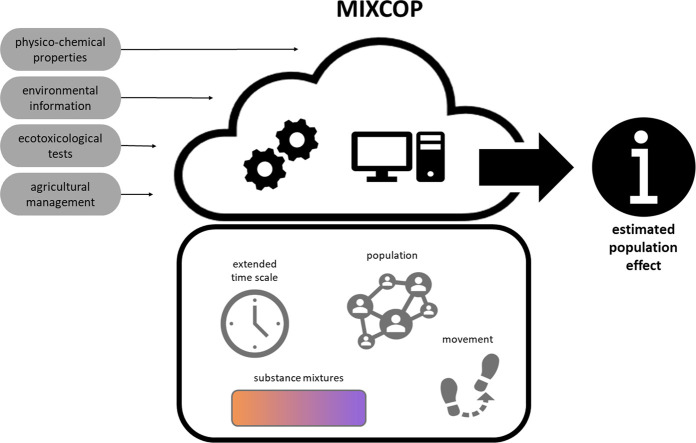

Specifications table

**Table:** 

Subject area:	Environmental Science
More specific subject area:	Ecotoxicology
Name of your method:	MIXCOP
Name and reference of original method:	MITAS [2], GUTS-RED [3], FOLCAS [4]
Resource availability	The R script implemented will be made available on GitHub following its publication: https://github.com/alexsyb/thesis

## Background

The MIXCOP model (pesticide MIXtures and their effects on a COllembolan Population) aims to assess the impacts of time-dependent pesticide mixtures on a collembolan population (*Folsomia candida*) by linking existing modelling approaches. The final model provides a rapid assessment of complex pesticide exposure scenarios, which otherwise can only be investigated in elaborate and costly experiments. Most of the following sections were published in advance in Sybertz [1].

## Method details

**Fig. 1:**
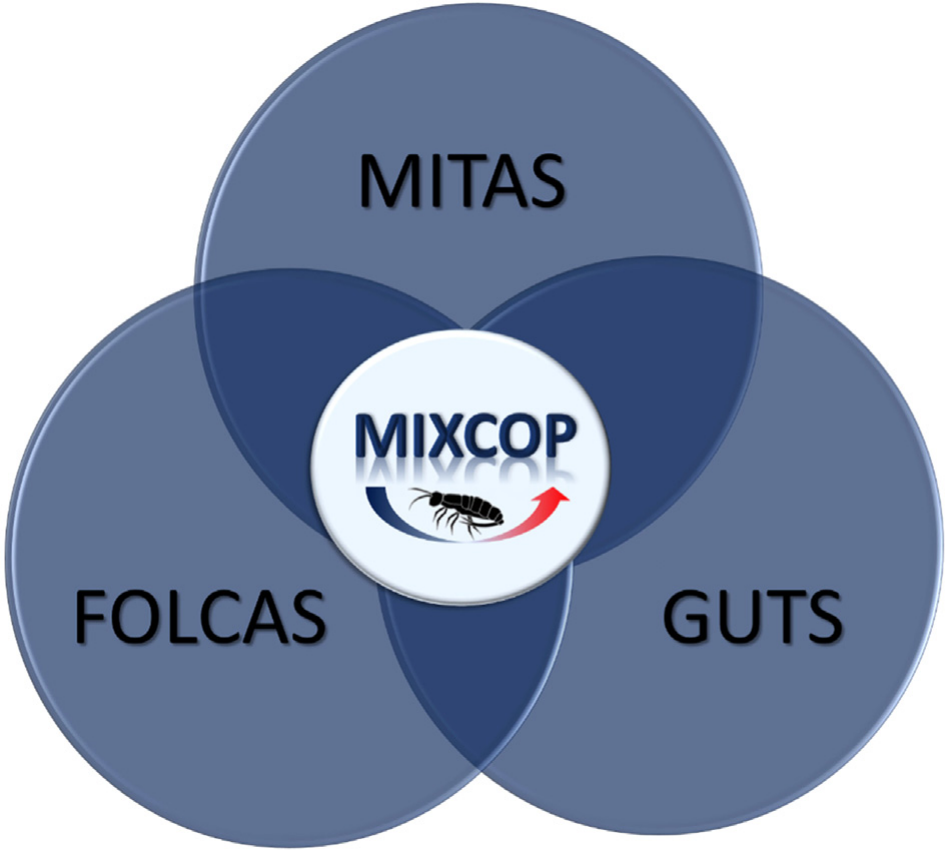
**General model concept**. The model is built from a combination of three individual models (MITAS, GUTS, FOLCAS). This graphic was published in advance in Sybertz [1].

Three different models are used to combine exposure, toxicity and population effects in the MIXCOP model (Fig. 1).

The complexity of pesticide exposure and effects led to the decision to combine three already existing models. The MITAS model is used to calculate pesticide exposure over time [2]. Calculation of the time-dependent toxicity is implemented by General Unified Threshold Models of Survival (GUTS)-modelling [5]. The FOLCAS-model [4] incorporates the population effects. It is an individual-based vertical distribution model for *Folsomia candida* [4].

**Fig. 2:**
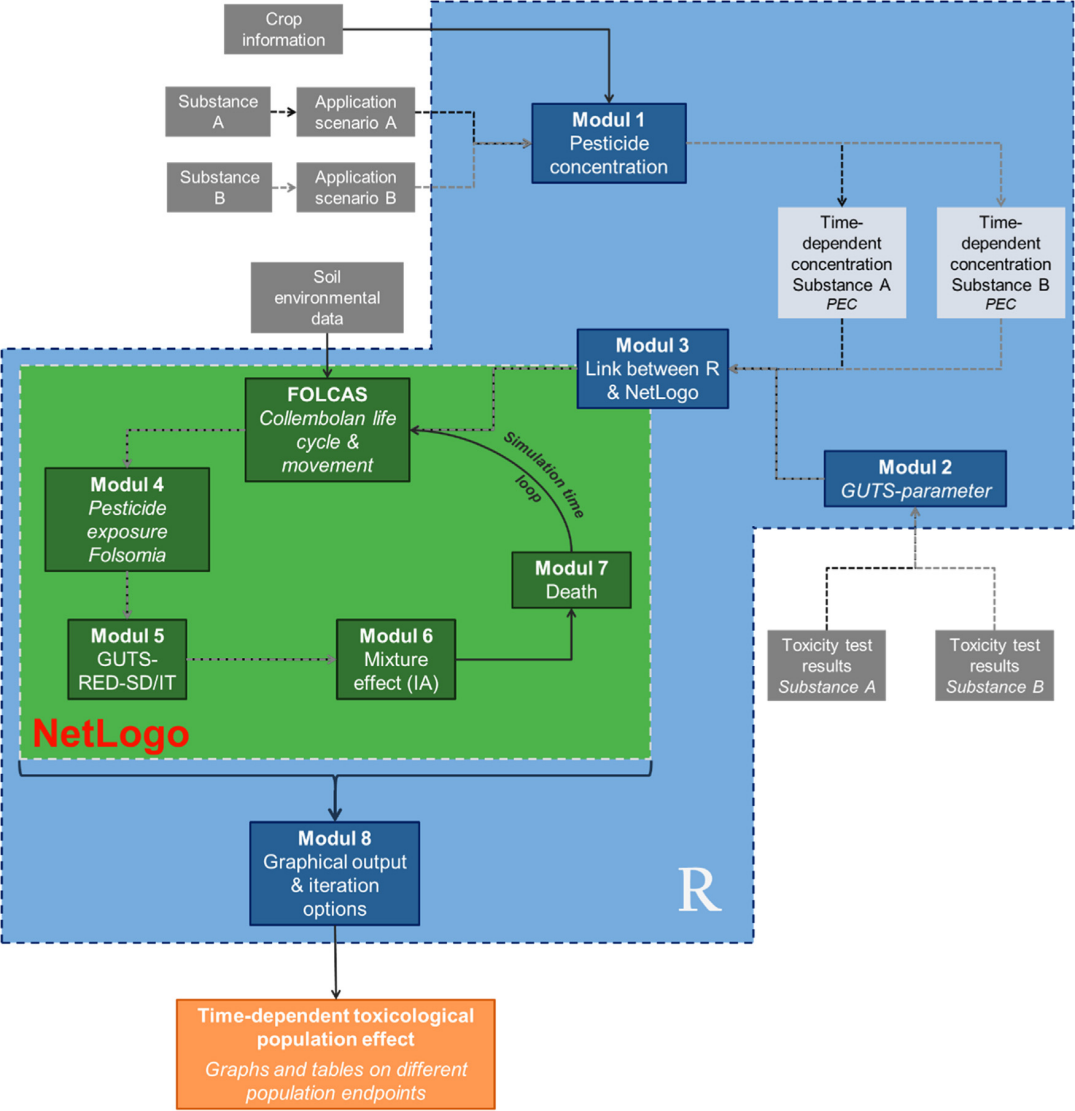
**Modular structure of MIXCOP**. Modules in the blue area are calculated or executed in R and modules in the green area are calculated in NetLogo. Calculations with single substances are shown with dotted arrows and solid arrows illustrate calculations with substance mixtures. Input data are displayed in grey boxes and output in orange boxes. This graphic was published in advance in Sybertz [1].

The overall concept of MIXCOP is modular and starts with the linkage of two different programming languages, R [6] and NetLogo [7], which was realized via a special R package called RNetLogo [8]. It enables control and data transfer between the two programming languages. To allow a flawless communication between the individual models a standardisation of the underlying environmental parameters and the applied calculations was done. R is used as common control system for the entire model. The modular structure enables the implementation of partial calculation steps, which ensures better control of the individual calculations (Fig. 2).

### Module 1: Pesticide concentration

Based on the specified pesticide application scenario the time-dependent PEC value (Predicted Environmental Concentration) in soil is calculated for each individual substance. The calculation is based on the model MITAS [2]. Same substances, which are applied at different time points, are combined in the calculation. The results are stored in a separate file, which is accessible and readable by NetLogo [7].

### Module 2: GUTS-parameter

The toxicological effect is calculated with a GUTS model. It enables the prediction of an effect, even if the applied substance concentration was not tested. GUTS models are based on the internal substance concentration in the organism (toxicokinetik=TK) and the process causing the effect (toxicodynamic=TD) [9]. Due to lack of detailed data for internal substance concentrations of the collembolan *Folsomia candida*, only reduced-GUTS (GUTS-RED) was applied in the MIXCOP model. It enables an effect prediction, despite a data shortage. Derivation of the GUTS parameters is implemented using the R-package morse [10]. These parameters are necessary for the final GUTS calculation in MIXCOP module 5. The effect prediction requires toxicological information for each applied substance for the collembolan (*Folsomia candida*), including replicates, tested concentrations, individual time points and number of surviving individuals.

### Module 3: Link between R & NetLogo

The implemented FOLCAS model [4] runs in NetLogo and refers to agricultural fields. It assumes a 2D soil column, which is horizontally homogenous and vertically heterogeneous. Various environmental parameters of the soil column affect the movement pattern of the simulated collembolan *Folsomia candida*. It is determined by the species’ preferred range with respect to the various abiotic parameters and the food offered. FOLCAS includes three developmental stages of the organism: egg, juvenile and adult. The R-package RNetLogo [8] is used to link the two modelling languages R and NetLogo. The FOLCAS model opens in module 3 and associated parameters are adjusted depending on the previously specified simulation scenario. In addition, the necessary information from module 1 and 2 are transferred to NetLogo. The following modules 4–7 run in the FOLCAS model within two loops, one for the selected number of simulation days (Fig. 2) and one for each individual collembolan.

### Module 4: Pesticide exposure

The individual exposure of the adult and juvenile collembolans is calculated in module 4. It depends on the substance concentrations (PEC values) already calculated in module 1. Pesticides are only present up to a certain soil depth in the model. The individual movement pattern of the collembolans at different soil depths in the FOLCAS model [4] simultaneously leads to unique pesticide exposure patterns for each organism during the simulation time. The exposure information is collected over time and stored in various variables and lists.

### Module 5: GUTS-RED-SD/IT

Reduced-GUTS (GUTS-RED) [5,11] is implemented to predict the potential toxicological effect of the applied pesticides. It is used because measured data on the kinetics of the compound and its uptake into the organism are not available or only very rarely available for *Folsomia candida*. For the calculation of the effect only the predicted GUTS-RED parameters are used from the R package morse [10] (module 2), the rest of the calculation consisting of toxicokinetics and toxicodynamics is done using a manual extension of the FOLCAS model. There are two assumptions to calculate the toxicodynamic, which describe two ways how the damage, caused by the substance, affects the survival rate [5]:

• Stochastic Death (SD) assumes all individuals are equal and if a certain threshold of damage has been exceeded, the probability of death increases with increasing damage.
• Individual Tolerance (IT) assumes a certain variability in the sensitivity of organisms. As soon as the damage exceeds the individual threshold of an organism, it dies.

Toxicokinetics are calculated the same for both assumptions.

The scaled internal concentration C_i_*(t) is calculated equally for both IT and SD based on Eq. (1) from Ashauer et al. [12], where k_e_ is the dominant rate constant and C_s_(t) is the external concentration in soil:

(1) $$\frac{C_{i}^{*}\left(t\right)}{dt}=k_{e}\left(C_{s}\left(t\right)-C_{i}^{*}\left(t\right)\right)$$

Eq. (1): Scaled internal concentration

The difference between the two assumptions is the calculation of toxicodynamics. The previously calculated scaled internal concentration is used for the calculation of the hazard rate h_z_(t) (Eq. (2)), which is the instantaneous probability to die. It is necessary for the calculation of the survival probability S_SD_(t) of the GUTS-RED SD (Eq. (3)). Those equations (Eqs. (2) & (3)) are also based on Ashauer et al. [12], where k_k_ is the killing rate constant and z the threshold concentration. Calculation of the survival probability for GUTS-RED IT S_IT_(t) (Eq. (4)) is again based on the equation of Ashauer et al. [12], where α is the median of the distribution and β is the shape parameter of the distribution.

(2) $$h_{z}\left(t\right)=k_{k}\max\left(0,C_{i}^{*}\left(t\right)-z\right)+h_{b}$$

Eq. (2): Hazard rate

(3) $$S_{SD}\left(t\right)=e^{-H_{z}\left(t\right)}$$

Eq. (3): Survival probability (SD)

(4) $$S_{IT}\left(t\right)=\left(1-\frac{1}{1+{\left(\max_{0\leq \tau \leq t}C_{i}^{*}\left(\tau \right)\;/\;\alpha \right)}^{-\beta }}\right)e^{-h_{b}t}$$

Eq. (4): Survival probability (IT)

The background hazard (h_b_) is assumed to be zero, due to the already included density dependent mortality in the population model [4]. Otherwise, the normal organism mortality would be overestimated.

### Module 6: Mixture effect (IA/CA)

Calculation of the mixture effect is done if at least two different substances are applied in the simulation. Two possibilities to calculate mixture effects are implemented: independent action (IA) [13,14] and concentration addition (CA) [15].

• The principle of independent action (IA), described by Bliss [13], predicts the mixture toxicity of substances with dissimilar mode of action [14]. IA assumes that compounds with different mode of action can only affect the part of the population, which is not already affected by another compound [16,17]. Effects of the single compound are added because each compound has an effect on the organism. Shared toxicity of the compounds is subtracted from overall toxicity. This method is based on the assumption that different modes of action cannot have shared effects [18,19].
• Concentration addition (CA) [15] assumes mixtures to consist of non-interacting compounds with same mode of action. Berenbaum [20] enhanced the concept of concentration addition with the aspect of multi-component mixtures [18]. In this model, effects rather than concentrations are summed.

### Module 7: Death

The module determines whether a collembolan dies. Each collembolan owns a variable that contains a random number between 0 and 1. This random number is compared with the previously calculated toxicity-driven mortality probability. This comparison is responsible for deciding whether the respective collembolan dies or not. If the mortality probability exceeds the random number, the collembolan dies. An additional variable is added to track the substance-related deaths. It counts the daily deaths of collembolans due to the applied substance. If only one adult collembolan remains, it does not die (refugium).

### Module 8: Graphical output

To improve evaluability, the results of the simulation are graphically processed with R. The R package ggplot2 is used for this [21]. During the simulation, data on the number of adult and juvenile collembolan, as well as the number of eggs present are collected and stored for each day. Additional information on the daily death rate of the collembolans, caused by the applied substance and the depth at which the adult and juvenile collembolan reside are also stored. These data enable a visualization and evaluation of the respective endpoints over the entire simulation period. It is possible to automatically repeat the simulation multiple times as an option.

One model endpoint is the total occurrence of adults, juveniles and eggs. This value represents the daily occurrence of individuals throughout the respective simulation time. It is important to note that this value does not reflect the total number of collembolans. Instead, each individual adult is counted for each day of its existence; for example, a single adult that exists for 10 days will contribute with 10 to the occurrence value. Nevertheless, the total occurrence is a good comparable measure for the existing individuals and their lifespan.

## Model application

To ensure the usability of the MIXCOP model, the computational speed of the final model is essential. This speed is influenced by the computational settings, as well as the number of applications and the available collembolans. A simulation run involving a substance with two applications over a duration of 365 days requires a minimum time investment of approximately 1.5 min. Increasing the number of iterations may result in a time requirement of around 3 min for three iterations. The model facilitates longer simulations to be conducted in a relatively short period. Some parameters of the MIXCOP model allow for changes or customization. Simulation settings that can be customized in MIXCOP are:

• Individual name for the modelled scenario
• List of the applied pesticide substances together with their application rates
• BBCH stage of the crop
• A potential reduction factor for the degradation rate
• Soil mixing depth
• Simulation time
• GUTS-assumption
• Number of iterations
• Mixture assumption
• Inclusion of juvenile mortality

## Method validation

To verify the implemented simulation in the previously presented MIXCOP model, a verification was conducted. Due to the complexity of the final MIXCOP model, an overall validation of the model was not possible (Table 2). Individual aspects of the model could only be recreated with a complex experimental test system. The movement profile of the FOLCAS model has already been calibrated in a trial [4,22].

## Exposure verification

Common assumptions of the German plant protection product approval are the basis of the MITAS model. For verification of the underlying calculation, the PEC calculation of MIXCOP was compared with the results of ESCAPE 2.0 (Estimation of Soil Concentration After PEsticide applications) [23], which was developed by the Fraunhofer IME in Schmallenberg, Germany. For the comparison, a single application of Teflubenzuron with an application rate of 0.6 kg/ha on day 1 was assumed. As environmental scenario, the bulk density was set to 1.3 g/cm^3^ with a constant temperature of 20 °C for better comparison. Degradation rate of the substance (DT50) was assumed to be 92.1 days [24,25]. PEC values for two different mixing depths were calculated: 5 cm and 20 cm. Calculations included time-dependent PEC-values in soil with single first-order degradation (Table 1).

**Table 1:** Time-dependent PEC-values calculated with ESCAPE 2.0 and MIXCOP.

Time [days]	Mixing depth: 5 cm		Mixing depth: 20 cm	
	ESCAPE 2	MITAS	ESCAPE 2	MITAS
**0**	0.9231	0.9231	0.2308	0.2308
**1**	0.9162	0.9162	0.2290	0.2290
**2**	0.9093	0.9093	0.2273	0.2273
**4**	0.8957	0.8957	0.2239	0.2239
**7**	0.8757	0.8757	0.2189	0.2189
**14**	0.8308	0.8308	0.2077	0.2077
**21**	0.7881	0.7881	0.1970	0.1970
**28**	0.7477	0.7477	0.1869	0.1869
**42**	0.6729	0.6729	0.1682	0.1682
**50**	0.6336	0.6336	0.1584	0.1584
**100**	0.4349	0.4349	0.1087	0.1087

Calculation is based on a single application with Teflubenzuron. The table shows the calculated PEC values in mg/kg soil for both models. The simulation was performed with two different soil mixing depth: 5 cm and 20 cm.

**Table 2:** MIXCOP verification.

	Exposure calculation	Effect calculation	Collembolan movement	Entire MIXCOP model
**Verified**:	☑ Table 1	☑ Figs. 3 & 4	☑ Roeben [4]	☒

Check marks are used for model parts already verified. The movement calibration and verification was already conducted by Roeben [4]. This table was published in advance in Sybertz [1].

Both the initial PEC values (day 0) and the consideration of the degradation were consistent in both models. The similarity of the results is due to same input parameters and the uniform underlying calculation assumptions for the PEC-calculation and the substance degradation. The calculation of these values is based on a purely deterministic model without stochastic assumptions.

## GUTS-RED verification

The correct implementation of the GUTS-RED modelling in MIXCOP was verified through a model comparison. GUTS-RED modelling is a well investigated method for predicting toxicity effects [26]. EFSA PPR Panel et al. [9] already declared GUTS as appropriate approach within the aquatic risk assessment of pesticides. A consistent implementation of the GUTS-RED model was ensured by using a computational comparison. Calculation of the effect is based on the input parameters of the morse package and the equations 1 - 4. This calculation option was referred to as “GUTSmanual”. The prediction was compared with results calculated entirely with the R package morse [10] (here referred to as GUTSmorse). Furthermore, supplementary calculations were conducted using openGUTS 1.2 [27] to ensure comparability independent of the morse package. A Teflubenzuron application with toxicological data from Xie et al. [28,29] was assumed for the comparison of the three GUTS-RED calculation options. To derive the exposure, an application rate of 0.6 kg/ha with a soil mixing depth of 5 cm was assumed in sugar beet. The resulting survival probability of the GUTS-RED calculations were compared with each other. Both individual tolerance (IT) and stochastic death (SD) were used as assumptions.

**Fig. 3:**
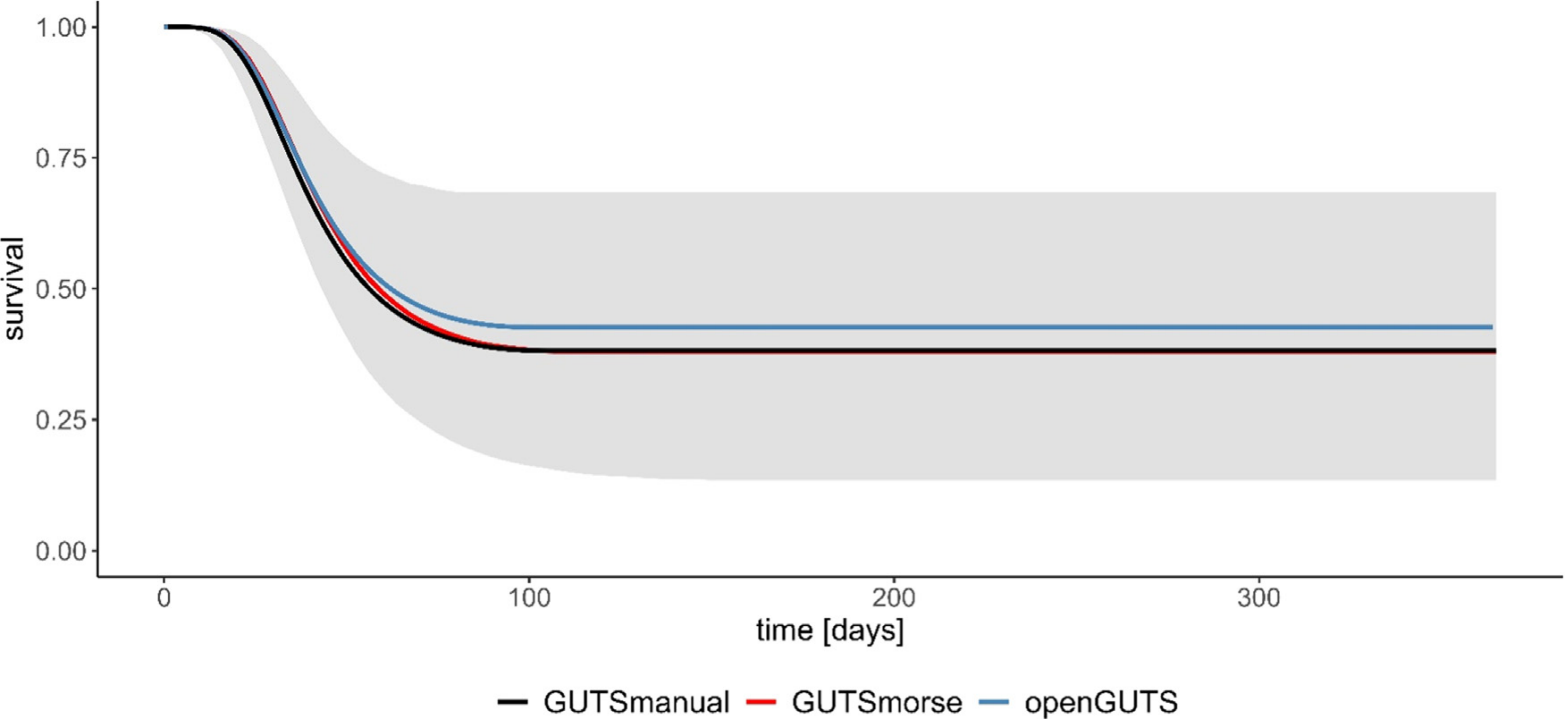
**GUTS-RED calculations with various models (individual tolerance)**. All graphs display the time course (days) on the x-axis and the respective survival probability (0–1) on the y-axis. The different colours correspond to the applied GUTS model. The grey area shows the credible interval of the morse calculation. The toxicity data on Teflubenzuron are obtained from Xie et al. [28,29].

**Fig. 4:**
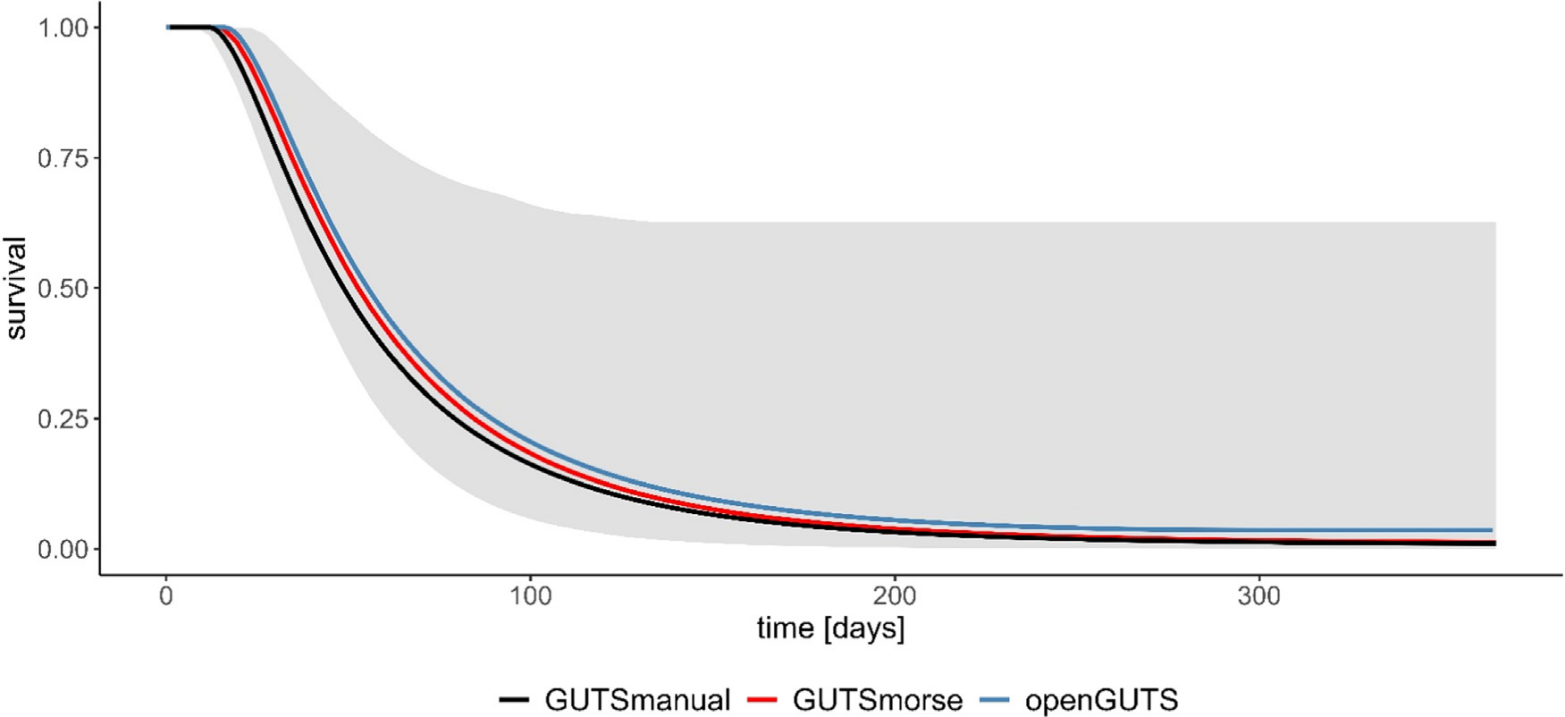
**GUTS-RED calculations with various models (stochastic death)**. All graphs display the time course (days) on the x-axis and the respective survival probability (0–1) on the y-axis. The different colours correspond to the applied GUTS model. The grey area shows the credible interval of the morse calculation. The toxicity data on Teflubenzuron are obtained from Xie et al. [28,29].

**Fig. 5:**
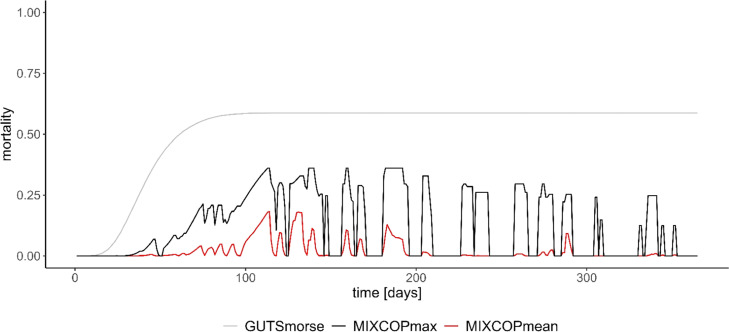
**Comparison of the effect calculation without the population model (GUTSmorse) and with the population model (MIXCOP) for one application of Teflubenzuron (individual tolerance)**. The time course is displayed on the x-axis and the mortality probability (0–1) on the y-axis. The calculation for MIXCOP shows the maximum (black) and mean (red) value from 3 runs. The toxicity data on Teflubenzuron are obtained from Xie et al. [28,29].

Compared to the established GUTS models, the implementation in the MIXCOP model appears to be appropriate (Figs. 3 & 4). The curve for both assumptions is comparable for all three GUTS models. This ensures a correct integration of the GUTS-RED calculation. Application and comparison of the two assumptions (SD & IT) shows clear differences in the results. SD calculates a continuous decrease of the survival probability to a value of 0 (Fig. 4). With IT, on the other hand, survival probability decreases only to about 0.4 and does not decrease any further (Fig. 3). In addition, a comparative analysis was conducted on the effect calculation, examining its results both with and without the population model (Fig. 5).

The comparative analysis, including and excluding the population model FOLCAS [4], shows a reduction of the mean mortality effect due to the population model (Fig. 5). The movement of the collembola in the model leads to a reduction in exposure and thus explains the decrease of the mortality probability through the integration of the population model. Nevertheless, the course of the maximum mortality probability of the MIXCOP model resembles the course without the population model. This acts as plausibility check to ensure the correct linkage of effect calculation and population model.

## Limitations

MIXCOP is quite user-friendly for people who have basic knowledge of R use. The complete model starts by executing an R script [6]. All information for an individual simulation scenario needs to be entered in a CSV file. Combination of these models increases the complexity of the entire model and enables the assessment of effects through the soil movement of organisms together with population development. Certain soil parameters within the model can even be adapted to the respective simulation scenario. Given sufficient data, this allows the direct comparison of time-dependent collembolan effects of two similarly applicable spray sequences using different substances. In combination with retrospective pesticide monitoring, MIXCOP can contribute to enhancing the protection of soil organisms in the long term. In general, the model facilitates a more comprehensive utilisation of the available toxicological data and provides additional insights into potential population-level effects. However, it should be noted that the model is predicated on data derived from laboratory species in artificial soil environments. Consequently, ecological interactions with other soil organisms and the impacts of collembolan reduction on the overall soil community are not accounted for within this framework.

**Model benefits:**

√ **Individual-based substance exposure**:
  √ Pesticide application scenario
  √ Temperature-dependent pesticide degradation
  √ Collembolan movement
√ **Time-dependent mixture effect**
√ **Collembolan life cycle**
  √ Different developmental stages
  √ Population impacts
  √ Indirect effects

While important toxicity and population aspects are already incorporated into the MIXCOP model, there are still useful elements missing. It should be noted that the model currently focuses on mortality as an endpoint, which could potentially lead to an underestimation of the overall effect. In order to incorporate further effects, such as effects on fertility or avoidance behaviour, in future, adequate data regarding collembolan are necessary. The optimal use of the MIXCOP model is to compare two potential spray sequences with different substances or application times to determine which one has a lower impact on collembolan populations. This approach enables adjustments to the spray sequence; utilizing data that are already available.

## Conclusion

MIXCOP is primarily an assessment support tool for considering pesticide mixture impacts on collembolan populations over a longer period of time. Furthermore, it allows for an integrated assessment of the potential ecological risk caused by agricultural practice. Each juvenile and adult collembolan is exposed to a unique pesticide exposure pattern that emerges through the application scenario, substance degradation and individual movement. This distinctive pesticide pattern serves as basis for the individual effect assessment and the development of the entire population.

## CRediT authorship contribution statement

**Alexandra Sybertz:** Conceptualization, Methodology, Software, Validation, Writing – original draft, Visualization. **Richard Ottermanns:** Conceptualization, Methodology, Writing – original draft. **Martina Roß-Nickoll:** Conceptualization, Methodology, Writing – original draft.

## Declaration of competing interest

The authors declare that they have no known competing financial interests or personal relationships that could have appeared to influence the work reported in this paper.

## Data Availability

The R script implemented will be made available on GitHubhttps://github.com/alexsyb/thesis
